# Relationship between saliva and blood cortisol in handled cows

**DOI:** 10.5713/ajas.18.0151

**Published:** 2018-07-26

**Authors:** Melody Dzviti, Lizwell Mapfumo, Voster Muchenje

**Affiliations:** 1 Department of Livestock and Pasture, University of Fort Hare, P. Bag X1314, Alice, 5700, South Africa

**Keywords:** Animal Stress, Behaviour Scores, Cows, Cortisol, Glucocorticoids

## Abstract

**Objective:**

The objective of the study was to determine the relationship between plasma and salivary cortisol concentrations in beef cattle that were subjected to handling prior to sampling.

**Methods:**

Twenty-one Nguni cows of three age categories; 5 to 7 yr (n = 7), 8 to 10 yr (n = 6), and 11 to 13 yr (n = 8) were handled for five consecutive weeks. In the pen, a human avoidance test was performed and cattle responses to restraint in the chute and crush were observed. In addition, rectal temperature readings were taken and, faecal samples were collected and analysed for glucocorticoid metabolites. Through the handling and restraint process, excretory and vocalisation behaviour, as a sign of stress were observed and recorded. Thereafter, six cows were randomly selected and subjected to an adrenocorticotropic hormone (ACTH) challenge. Blood and saliva samples were extracted to determine cortisol concentrations.

**Results:**

Repeated handling affected (p<0.05) faecal glucocorticoid metabolites, rectal temperatures, avoidance distance, crush scores as well as urination and defaecation behaviour. Acclimation to handling was variable based on each respective parameter. Saliva cortisol concentrations increased and decreased significantly (p<0.001). A peak value of 136.78± 15.869 nmol/L was observed 30min after administration of ACTH, from a baseline value of 8.75± 15.869 nmol/L. Plasma cortisol concentrations did not differ (p>0.05) across the time of sampling. A low and insignificant correlation (r = 0.0131, p>0.05) between plasma and saliva cortisol was therefore observed.

**Conclusion:**

We conclude that if beef cows are subjected to handling prior to sampling, a weak relationship exists between plasma and salivary cortisol levels.

## INTRODUCTION

Human beings have become more sensitive to the welfare of farm animals. For each individual animal, the quality of life contributes to productivity, with better welfare promoting increased efficiency compared to poor welfare. Animal well-being can however be depressed during aversive human-animal interactions [1] that are done in the pens and crushes. These handling facilities are designed for management practices such as vaccinations that enhance animal welfare. However, they are usually associated with the isolation of an individual animal from its cohorts, for ‘aversive’, stressful and fearful handling [2]. It is therefore maintained that in handling facilities such as the race and crush, cattle lose control and this in itself can be stressful. Hence, race and crush scores are some of the indices that have since been used to determine the extent to which animals can actively resist or be willing to be restrained. Eccentric behaviour, such as stupor, restlessness, kicking, shaking the facility and attempt to escape can be displayed by animals that are reluctant to be handled and restrained [2].

Behavioural responses such as avoidance, and the distance maintained from handlers can be used to determine fear of cattle towards humans. The avoidance distance (AD) test has since been used to determine the distance at which animals withdraw from an approaching human [3]. Further, excretory behaviour such as urination and defaecation can be frequent especially in fearful animals [4]. Repeated exposure of an animal to the same stressor is however associated with reduced subsequent responses [5], such that aggressive animals that regularly go through the same handling procedures may progressively become docile.

Animal handling and restraint on the other hand trigger physiological responses such as the activation of the hypothalamic-pituitary-adrenal (HPA) axis exhibited in increased release of glucocorticoids (GCs) such as cortisol. Cortisol is therefore used to quantify stress [6]; hitherto, blood cortisol is often used as a reliable stress biomarker, to determine how animals respond to different degrees of stress [7]. Cook [8] however highlighted that handling may have confounding effects on the measurements, which can be caused by restraint and venepuncture. In addition, Rushen [9] underlined that data related to the HPA is inconsistent. To mitigate erratic measurements, less-invasive procedures which make use of saliva and faecal samples are being used.

For more than a decade, the quantification of faecal GCs has become popular as a non-invasive tool in the study of adrenocortical activity during stressful situations. Faecal glucocorticoid metabolites (FGM) are used for the measurement of anthropogenic disturbances on animals [10] and in cattle, the use of dung to reflect faecal GCs is validated [11]. To our knowledge, the relationship between FGM and stress-related behaviour is illustrated in some zoo and wild animals such as the marmoset [12], as well as the domestic horse [13], but is not yet determined in cattle. Observation of animal behaviour in relation to handling stress is less-invasive. Nevertheless, accurate assessment of how cattle react to handling and restraint must employ both behavioural and physiological parameters [14].

The juxtaposition of behaviour and physiological parameters can be used to assess animal welfare. For instance, rectal temperature, a more reliable parameter for measuring core body temperature, increased in cattle that exhibited adverse behavioural reactions during restraint in the chute [15]. Understanding of behaviour and physiological parameters can help reduce animal stress and ultimately increase animal production efficiency. High GC values can be expected in animals that lack experience in handling stress. However, as the number of handling encounters increase, the magnitude of GC levels can be anticipated to subsequently decline [5].

Salivary cortisol reflects the biologically active fraction of the total plasma steroid hormone with a positive relationship between the two media [16]. Moreover, the association between the media is linear over a wide range of concentrations [8,16]. Saliva samples can be collected both prior to and after an imposed stress even at fixed time intervals [17], to indicate the activity of the HPA axis in response to different stressors. The correlation of plasma and saliva cortisol has been established in some domesticated ruminant animals such as goats [18,19] and sheep [20,21]. Studies that validate the use of saliva cortisol as an alternative to blood cortisol in cattle are however still limited. Hitherto, a hormonal challenge was done based on sampling intervals of at least 15 min [17,22]. The study by Hernandez et al [23] was characterised by short sampling intervals of 10 min, and, the hormonal challenge was not done. Notwithstanding, plasma cortisol levels can change due to non-aversive procedures. Thus, cattle handling prior to sampling can affect the characterisation of the relationship between plasma and saliva cortisol. Our objective was to determine the relationship between plasma and salivary cortisol levels in ACTH induced beef cows that were handled prior to sampling. Acclimation to handling was done prior to the characterisation of the relationship.

## MATERIALS AND METHODS

### Ethical approval of animal use and location

All procedures involving animals were reviewed and approved by the University of Fort Hare Animal Research and Ethics Committee (Reference Number: MUC291SDZV01). The study was conducted at a research farm under the coordinates 32°48′S (Latitude) and 26°53′E in Alice, South Africa during mid-spring (mean temperature 23°C±4.1°C).

### Description and management of animals

Twenty-one clinically healthy non-pregnant Nguni cows aged between 48 and 144 months and weighing between 426 and 436 kg were selected at random, from the different camps at the farm. They were of different age groups, group 1 with n = 7 (5 to 7 yr), group 2 with n = 6 (8 to 10 yr), and group 3 with n = 8 (11 to 13 yr) and these had average weights of 436 kg, 429 kg and 426 kg for each respective age group. The cows were identified by ear-tagging and markings on the flanks according to the respective farm’s management system of identification. The identification system made it feasible to use the same cows throughout the trial. The cows were handled separately, however, they were all exposed to handling through routine paddock rotations, health and welfare related check-ups. All the cows had access to grazing on natural pastures and water *ad libitum*.

### Measurements and data collection

#### Experiment 1

**Figure 1:**
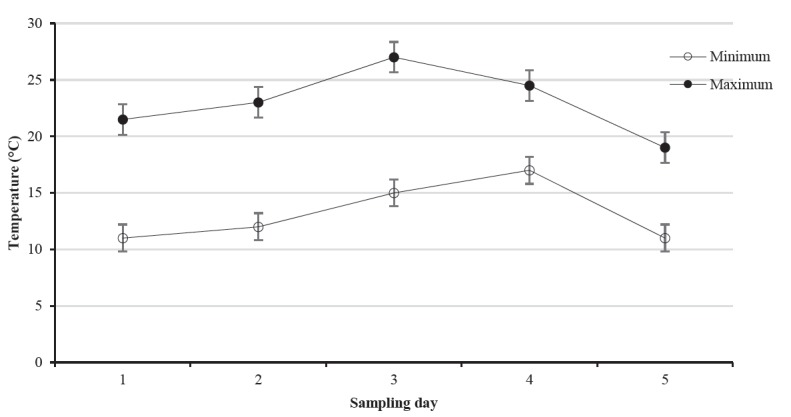
Line graphs showing the average maximum ( 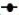) and minimum ( 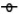) environmental temperatures during the five sampling days for Experiment 1. The temperatures increased then decreased as indicated by the patterns of graphs and error bars represent standard error of means.

Data was collected from each individual animal unsystematically between 9:00 and 11:00 am from July to September. There was a seven-day interval per observation and sampling per animal. Maximum and minimum temperatures during sampling days are indicated in Figure 1.

Different animal groups were separately brought into the holding pens 14 h prior to observations and samplings. The AD test was done on each randomly selected individual animal in a handling pen, based on modifications to the studies by Dodzi and Muchenje [3]. The handler who wore the same clothing (green work-suit) on every occasion, approached an individual stationary cow at a rate of one step every 5 s from a distance of 3 m, whilst avoiding direct stares to the animal. The test ended when the cow showed an avoidance reaction (retreat). Scores 1 to 3 as indicated in Table 1 were allotted accordingly. The lower end indicated that the animal was less willing to be approached by the handler than the one which was assigned a Score 3. For cows which could not be immobile enough to be approached for the AD test, an AD Score 1 was assigned.

Each individual animal was then restrained in the chute for 30 s and its behaviour observed and scored based on scores modified from Goldhawk et al [24]. Thereafter, for 15 s the cow was restrained in the crush pen with its head fixed with a head gate. Immediately after fixation, behaviour was observed and evaluated with the scoring system in Table 1.

Rectal temperature readings were then collected on the assumption that, human contact affects cattle behaviour and this may stimulate different biological variables including the former [15]. A digital thermometer (GLA M500, GLA Agricultural Electronics, San Luis Obispo, CA, USA) was used and readings recorded as done by Sánchez-Rodríguez et al [25]. Ambient temperature readings were also taken. Fresh faecal samples were then extracted directly from the rectum of each cow, using a gloved hand and placed in sterile vials. The samples were placed on ice and thereafter frozen at −20°C until analysis.

During handling, vocalisation, excretion of watery faeces and urine were observed. Only occurrences of at least three times during the sampling times were considered. The parameters were recorded and assigned scores as either present (1) or absent (0), thereby giving vocalisation, defecating and urinating scores accordingly. From when avoidance behaviour was observed to when the animals were released from the crush pen, total handling time for each animal was on average 120±10 seconds.

##### Faecal sample analysis

Faecal samples were defrosted at room temperature (20°C to 23°C) over an average period of 4 h. A well-mixed (stirred) wet faecal sample (1 g) was dispersed in 5 mL of 80% methanol and vortexed for 16 h. Of the dispersion, 50 μmL was pipetted into Eppendorf tubes, centrifuged at 1,000×g for 10 min at 4°C and mixed into a methanol 4:1 distilled water solution. The FGM concentrations were assayed using a commercial double-antibody 125I-corticosterone radioimmunoassay kit for animal testing (MP Biomedicals, 07120103, Carlsbad, CA, USA; Lot No. RCBK163) at half volume [26]. The supernatant was aspirated and the precipitate counted on a PerkinElmer Wizard2 Gamma Counter. The intra-assay coefficient of variation was 7.1%.

#### Experiment 2

- Animals: Six clinically healthy non-pregnant Nguni cows were randomly selected from animals used for experiment 1. The cows had a mean weight of 407 kg (range: 336 to 506 kg) and each cow was used as an experimental unit. Samples were collected between 08:30 and 11:30 h at ambient temperatures of not more than 30°C (Figure 1).
- Adrenocorticotropic hormone administration: A standardised dose (1 μg/kg), of ACTH (Synacthen Depot, tetracosactide 1 mg/mL, Lot S1358, Novartis, South Africa) was administered once to each of the six cows at time 0 [27].
- Extraction of blood samples: Blood samples (approximately 6 mL) were collected whilst the animals were restrained in a crush pen with the head in a head gate through jugular venepuncture into tubes containing SST gel. A nose grip was also used for stable restraint. Baseline samples were collected 10 min prior to ACTH administration. Thereafter, samples were drawn every 10 min for 1 h [28], to obtain a total of eight samples per animal. Samples were placed on wet ice (4°C) and then centrifuged for 10 min at 3,550×g (22°C). The serum was then transferred to red-topped tubes (4 mL) with a Clot Activator and stored at −20°C until analysis for cortisol.
- Extraction of saliva samples: Saliva samples were collected immediately after blood sample collection using cotton based swabs (Salivette cortisol; Sarstedt, Nümbrecht-Rommelsdorf, Germany), which provided a method for easy and safe collection. Cotton balls were also used to collect drooling saliva to maximise the amount of saliva collected. The cotton swabs were inserted at an angle of the lips into the mouths of the cows with the help of a nose grip until well soaked [29]. Each swab and the corresponding cotton ball were then placed in the salivette tube which was placed on ice (4°C). The samples were centrifuged at 1,000×g for 2 min at 20°C. The swabs and cotton balls were removed together with the inner tube of the salivettes. The saliva which collected into the outer tubes was immediately stored at −20°C until analysis. Salivary cortisol was determined by competitive enzyme-linked immunosorbent assay (ELISA).
- Cortisol analysis in plasma and saliva: Blood plasma samples were defrosted and vortexed (Vortex Genie-2, Scientific Industries Inc, New York, USA) at room temperature (24°C). Quantification of plasma cortisol was then done by ELISA using a Cortisol ELISA kit (IBL International, GmBH, Hamburg, Germany, RE52611) as described by Olbrich and Dittmar [30].

Saliva samples were defrosted and centrifuged at 7°C, 1,200 ×g for 10 mins and cortisol levels were measured as explained for plasma cortisol. The intra-assay coefficient of variation (CV) ranged from 3.1% to 6.1% for saliva, and 5.9% to 9.9% for plasma. The inter-assay CV ranged from 4.2% to 17.0% for saliva and 13.0% to 20.0% for plasma. The detection limits of the saliva and plasma assays were 0.083 nmol/L and 0.14 nmol/L, respectively. For both saliva and blood plasma, optical density measurements were done at 450 nm using a photometer Multiskan Ascent with a Genesis Lite microplate computer programme (Labsystem, Finland).

### Statistical analysis

#### Experiment 1

Data on subjective scores were square root transformed. Thereafter, all data on FGM concentrations, rectal temperature; avoidance, chute, crush, urinating, defecating and vocalising behaviour scores, were analysed using a repeated measure PROC general linear model procedure of Statistical Analysis System (SAS Inst. Inc., Cary, NC, USA). A repeated measures analysis of covariance with weight as a covariate, was run to determine the differences within variables. Week of sampling and age were fixed factors and animal was a random variable. Significant differences among means were tested by use of the Fisher’s least significant differences method at p<0.05. The model was differences were considered statistically different at p<0.05. The model used was as follows: Y_ijk_ = μ+α_i_+β_j_+γ(W*) +ɛ_ijk_; where; Y_ijk_ is the response variable (physiological and subjective parameters); μ is the mean; α_i_ is the effect of sampling week (i = 1, 2, 3, 4, 5); β_j_ is the effect of group (1, 2, 3); γ(W*) is the adjusted covariate mean and ɛ_ijk_ is the standard error. Pearson’s correlation coefficients were also determined using SAS.

#### Experiment 2

Data were analysed by means of a repeated measures general linear model of SAS. The individual cow was a random variable and time was a repeated factor. Data for plasma cortisol was adjusted for normality using the log-transformation. Significance was set at p<0.05 and values are given as means±standard error of the mean (SEM). To assess the relationship between plasma and saliva cortisol the CORR procedure of SAS was used.

## RESULTS AND DISCUSSION

### Experiment 1

The FGM were assayed and rectal temperatures were measured in addition to observing stress-related behaviour. The FGM and rectal temperatures were different throughout the sampling weeks (Table 2). Repeated handling, however affected the physiological parameters, avoidance and crush behaviour as well as excretory behaviour. The FGM can be estimated to quantify the level of stress an animal is subjected to. We anticipated that with each handling encounter, FGM values would decrease. From our findings, the values were erratic, however, they are generally comparable to those reported by Xavier et al [31]. When there is an overrepresentation of faeces of some individuals, FGM values can be inconsistent [32]. Our study was however characterised by individual identification of each faecal sample, making the source of variability relatively indeterminable. In the same way, we recorded variable rectal temperature values, though they were analogous to those reported by Gruber et al [15] (range: 38.3°C to 40.8°C). Restlessness as a result of movements associated with the experimental procedures could have triggered irregular blood flow thereby stimulating erratic rectal temperatures. In addition, variable responses of the different individuals could have contributed to erraticism. Furthermore, Grandin [14] highlighted that such inconsistencies in the physiological parameters can be attributed to fear, a psychological stressor.

Fear can be regarded as a reaction to imaginary danger [33], whereby an animal is fearful of that which is perhaps absent or non-existent. As cattle are gregarious animals, they are vulnerable to exhibit fear of novelty. Accordingly, the general isolation of each individual animal from its respective group for the avoidance test and restraint in the crush could have elicited such kind of fear, especially in the first weeks [4]. Fear of novelty or the human-animal interaction could have simultaneously triggered excretory behaviour in the first weeks [34]. Perhaps, this could be the reason why the Pearson’s correlation test, indicated a significant positive relationship between AD test and urination, though it was moderately weak (Table 3). However, we could not establish why excretory behaviour increased as animal behaviour became more docile while the animals were restrained in the crush. On the other hand, results imply that animals that avoid human approach at longer distances (<3 m away but ≥2 m away) have a tendency of being aggressive in the chute and the crush.

Results for the avoidance test and restraint in the crush showed some degree of animal adaptation to human approach and handling (Table 2). Animals can habituate if they can control or predict the stressor. The animals might have become familiar with the handler and the handling procedure, thereby becoming more at ease with being approached as indicated by a decrease in the ADs. In addition, cows could have learned that after restraint in the crush, they were to be released, thus elucidating calmer behaviour during the last weeks of handling.

We anticipated that group 1 cows would be more excitable during handling than their cohorts, assuming that they have the least human-animal and restraint experience [5]. Such behaviour was only observed whilst the cows were handled in the chute. Their behaviour was however not maintained through the other procedures and therefore we assume that cows can for one reason or the other, react variably even when subjected to uniform environmental conditions. On the other hand, group 2 cows excreted urine less than (p<0.05) the other cows (Table 2). This could imply that cows in this group were generally less stressed than their cohorts. However, resolute conclusions could be made if the other parameters showed a similar trend.

### Experiment 2

**Figure 2:**
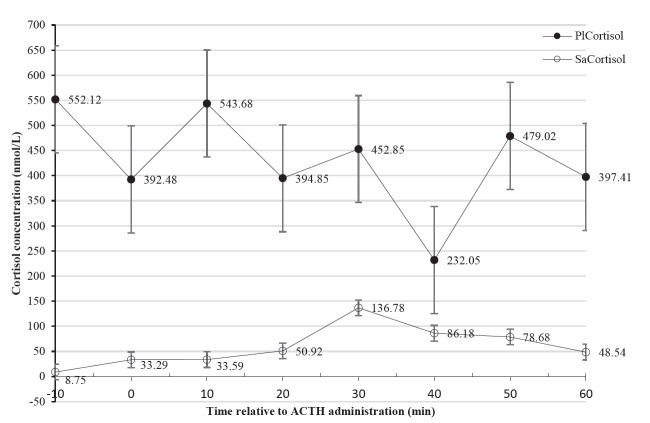
Cortisol concentrations of cows subjected to a hormonal challenge using a standardised dose (1 μg/kg) of adrenocorticotropic hormone at time 0. Line graphs represent plasma cortisol ( 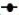 PlCortisol) and salivary cortisol ( 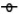 SaCortisol) concentrations. Line graphs represent cortisol concentrations which either increased or decreased as depicted by the down-up or up-down lines respectively and the error bars represent the standard error of means. Insignificant differences were observed for plasma concentrations in contrast to the saliva concentrations which were variable (p<0.001).

Figure 2 illustrates results for plasma and saliva cortisol. Data are presented as means±SEM.

Both plasma and saliva cortisol concentrations were expected to escalate after the hormonal challenge and decrease thereafter to baseline values. Basal plasma cortisol concentrations did not significantly increase (p>0.05) after ACTH administration (Figure 2). Endogenous secretion of ACTH could have occurred at its peak due to the handling involved in the study. In contrast, saliva cortisol concentrations significantly increased (p<0.05) 30 min after the challenge, suggesting that there is an association between the HPA axis and saliva cortisol. Similar findings for saliva were reported for dairy cows [17,22]. These early studies however observed lower baseline values for both plasma and saliva cortisol than those reported in this study. Schwinn et al [22] assayed saliva cortisol concentrations of about 2.79 to 19.45 nmol/L (converted) and 6.32 to 60.31 nmol/L for plasma cortisol whereas Negrão et al [17] reported baseline values of approximately 5.52 to 8.28 nmol/L for plasma cortisol. It was however stated by Dunn [34] that extreme stress would cause plasma cortisol values of 256.59 nmol/L (93 ng/mL) in cattle, suggesting that values obtained in our study (552.12±106.617 nmol/L, plasma cortisol and 8.75±15.869 nmol/L, saliva cortisol) were generally high.

Activation of the HPA axis begins during restraint and handling [7]. From the time when the animals were restrained in the pen to when they were in the crush pen, both plasma and saliva cortisol levels could have risen. Restraint itself stimulates a dramatic increase in cortisol [5], especially with respect to plasma, and this could have occurred in our study. The cows exhibited high avoidance behaviour as they at many occasions retreated from entering the crush pen; therefore, fear a psychological stressor might have contributed to elevated baseline values. High cortisol values are expected in animals that do not willingly enter a crush [5]. These could be reasons why basal plasma cortisol values were already high before hormonal stimulation. Repeated handling thereafter might have then led to unrestrained stress levels. It is also important to note that adaptation to stress depends on the intensity and type of the handling experience [3], hence, repeated venepuncture could have contributed to confounding effects on cortisol levels [8].

Total plasma cortisol increase is an indicator that an animal is exposed to stress. However, the free plasma cortisol fraction accounts for the exponential rise, thus defining the biological activity of plasma cortisol. Saliva sampling is done to determine cortisol levels as a reflection of the free plasma state [16]. In saliva cortisol, only the biologically active fraction is measured [7] and the steroid concentrations only reflect those in the free fraction of plasma. Results for this study, suggest that plasma cortisol could be a reflection of the total plasma cortisol fraction. This could have been coupled with its volatility making baseline values difficult to determine. In addition, response to stress is governed by the interplay of aggressive previous handling experiences and genetic factors such as temperament. Thus, cattle that are naturally excitable, have difficulty in adapting to handling procedures. These excitable cattle are associated with increased physiological responses [14]. This tallies with observations that cows used in this study displayed aggression such as shaking the chute during sampling.

Salivary cortisol is associated with a pronounced activation of the HPA axis and is correlated to plasma cortisol [22]. Our study, to a large extent, was consistent with the assertion indicated by the pattern for saliva cortisol. However, due to varying patterns in saliva and plasma cortisol upon ACTH challenge, we observed a weak insignificant correlation (r = 0.0131, p = 0.9310) between saliva and plasma cortisol concentrations. Thus, our results for the correlation were inconsistent to findings by other researchers [17,22,23]. Weak correlations between the two media were however reported in dairy cattle subjected to feeding and drinking actions [22]. In the same way, the delay to reach a steady state between plasma and saliva cortisol as suggested by Hernandez et al [23] was not observed in our study, consistent with the reports by Schwinn et al [22]. While the fact that the breed used is an aggressive breed, the predictor variables of the relationship between plasma and saliva cortisol could be a combination of independent and mediating factors such as causal activity. However, how animals respond and cope with handling or restraint, cannot be easily understood due to the complex physiological and biochemical responses involved [15], something to be considered in our study.

In conclusion, no significant relationship was observed between serum and salivary cortisol levels in beef cows that were handled prior to sampling. Cows that are fearful of being approached by handlers, can exhibit excitable behaviour in the crush, thereby influencing physiological response. This study can therefore be regarded as a preliminary for more studies in beef cows.

## Figures and Tables

**Table 1:** Avoidance distance, chute and crush scores used for this study

Category	Score
Responses to an approaching experimenter	Avoidance score
Animal avoided the approaching person at a distance <3 m away but ≥2 m away	1
Animal avoided the approaching person at a distance <2 m but ≥1 m away	2
Animal avoided the approaching person at a distance <1 m but ≥0 m away	3
Behaviour displayed in the chute	Chute score
No resistance to movement	1
Moves entire body to and fro rhythmically at least twice	2
Uneasy, head, body, tail and feet movements, hides head under another cow’s abdomen	3
Active escape behaviour	4
Rears and needs encouragement to move forward	5
Stupor/refusing to move requiring force to probe forward	6
Behaviour exhibited in the crush (modified from Geburt et al [28])	Crush score
Cow is calm with no movement	1
Cow is slightly excited with minimal movement	2
Cow is nervous and occasionally shakes the crush	3
Cow is agitated and continuously moves and shakes the crush	4
Cow is very agitated and exhibits escape behaviour, animals may kneel/fall	5

**Table 2:** Effect of sampling week and age category on the physiological and behavioural parameters1)

Variable	Sampling and observation week					SEM	Group			p-value		
		
	1	2	3	4	5		1	2	3	Week	Group	W×G
FGM	23.84^bc^	19.08^c^	30.57^a^	26.40^ab^	23.44^bc^	1.776	23.84±1.249	25.10±1.444	25.06±1.464	0.0008	0.5978	0.6350
Rect T	37.88^b^	38.76^a^	38.30^ab^	37.78^b^	38.12^b^	0.207	38.09±0.146	38.19±0.168	38.22±0.171	0.0052	0.8071	0.2400
ADS	1.20^b^	1.21^b^	1.38^a^	1.47^a^	1.51^a^	0.053	1.28±0.038	1.35±0.043	1.43±0.044	<0.0001	0.0841	0.1250
Chute	2.00	1.88	1.84	1.75	1.77	0.073	1.99^a^±0.052	1.78^b^±0.060	1.78^b^±0.060	0.1540	0.0084	0.3539
Crush	1.85^a^	1.87^a^	1.63^b^	1.56^b^	1.47^b^	0.067	1.67±0.047	1.58±0.054	1.77±0.055	<0.0001	0.0514	0.1829
Urin	1.02^b^	1.13^ab^	1.10^ab^	1.21^a^	1.21^a^	0.040	1.11^b^±0.028	1.22^a^±0.033	1.08^b^±0.033	0.0065	0.0045	0.8710
Def	1.14^b^	1.13^b^	1.17^b^	1.32^a^	1.24^ab^	0.044	1.19±0.031	1.23±0.036	1.18±0.036	0.0085	0.4836	0.1801
Vocal	1.09	1.10	1.17	1.15	1.17	0.045	1.10±0.031	1.21±0.036	1.10±0.036	0.5806	0.0531	0.9798

SEM, standard error of means; W×G, interaction of sampling and observation week and group; FGM, faecal glucocorticoid metabolites concentrations (ng/g); Rect T, rectal temperature (°C); ADS, avoidance score; Chute, chute score; Crush, crush score, Urin, urinating score; Def, defecating score; Vocal, vocalisation score.

1) Data are least squares (LS) means.

abc Means within a row without a common superscript significantly differ (p<0.05).

**Table 3:** Pearson correlation coefficients for parameters

Variable	FGM	RT	Avoidance	Chute	Crush	Urinating	Defecating
RT	0.102	-	-	-	-	-	-
Avoidance	−0.003	−0.024	-	-	-	-	-
Chute	−0.011	0.135	−0.372**	-	-	-	-
Crush	−0.125	−0.017	−0.351**	0.241*	-	-	-
Urinating	−0.022	−0.004	0.280*	−0.132	−0.335**	-	-
Defecating	0.099	−0.035	0.188	−0.117	−0.319*	0.156	-
Vocalising	−0.011	−0.077	0.067	−0.198	−0.077	0.127	0.027

FGM, faecal glucocorticoid metabolites concentrations; RT, rectal temperature; Avoidance, avoidance distance test score; Chute, chute score; Crush, crush score; Urinating, urinating score; Defecating, defecating score; Vocalising, vocalisation score.

* p<0.05,

** p<0.01.

## References

[1] Rushen J, de Passillé AMB, Munksgaard L (1999). Fear of people by cows and effects on milk yield, behavior, and heart rate at milking. J Dairy Sci.

[2] Kilgour RJ, Melville GJ, Greenwood PL (2006). Individual differences in the reaction of beef cattle to situations involving social isolation, close proximity of humans, restraint and novelty. Appl Anim Behav Sci.

[3] Dodzi MS, Muchenje V (2011). Avoidance-related behavioural variables and their relationship to milk yield in pasture-based dairy cows. Appl Anim Behav Sci.

[4] Müller R, Schrader L (2005). Behavioural consistency during social separation and personality in dairy cows. Behaviour.

[5] Grandin T, Shivley C (2015). How farm animals react and perceive stressful situations such as handling, restraint, and transport. Animals.

[6] Browning R, Leite-Browning ML (2013). Comparative stress responses to short transport and related events in Hereford and Brahman steers. J Anim Sci.

[7] Mormède P, Andanson S, Aupérin B (2007). Exploration of the hypothalamic-pituitary-adrenal function as a tool to evaluate animal welfare. Physiol Behav.

[8] Cook NJ (2012). Review: Minimally invasive sampling media and the measurement of corticosteroids as biomarkers of stress in animals. Can J Anim Sci.

[9] Rushen J (1991). Problems associated with the interpretation of physiological data in the assessment of animal welfare. Appl Anim Behav Sci.

[10] Touma C, Palme R (2005). Measuring fecal glucocorticoid metabolites in mammals and birds: the importance of validation. Ann NY Acad Sci.

[11] Palme R, Robia Ch, Messmann S, Hofer J, Möstl E (1999). Measurement of faecal cortisol metabolites in ruminants: a non-invasive parameter of adrenocortical function. Wien Tierarztl Monatsschr.

[12] Duarte RBM, Patrono E, Borges AC (2015). High versus low fat/sugar food affects the behavioral, but not the cortisol response of marmoset monkeys in a conditioned-place-preference task. Physiol Behav.

[13] Yarnell K, Hall C, Royle C, Walker SL (2015). Domesticated horses differ in their behavioural and physiological responses to isolated and group housing. Physiol Behav.

[14] Grandin T (1997). Assessment of stress during handling and transport. J Anim Sci.

[15] Gruber SL, Tatum JD, Engle TE (2010). Relationships of behavioral and physiological symptoms of preslaughter stress to beef longissimus muscle tenderness. J Anim Sci.

[16] Vining RF, McGinley RA, Maksvytis JJ, Ho KY (1983). Salivary cortisol: a better measure of adrenal cortical function than serum cortisol. Ann Clin Biochem.

[17] Negrão JA, Porcionato MA, de Passillé AM, Rushen J (2004). Cortisol in saliva and plasma of cattle after ACTH administration and milking. J Dairy Sci.

[18] Singh SP, Natesan R, Sharma N, Singh MK, Rahal A (2018). Lipopolysaccharide exposure modifies salivary and circulating level of cortisol in goats. Small Rumin Res.

[19] Greenwood PL, Shutt DA (1992). Salivary and plasma cortisol as an index of stress in goats. Aust Vet J.

[20] Fell LR, Shutt DA, Bentley CJ (1985). Development of a salivary cortisol method for detecting changes in plasma “free” cortisol arising from acute stress in sheep. Aust Vet J.

[21] Yates DT, Ross TT, Hallford DM, Yates LJ, Wesley RL (2010). Technical note: Comparison of salivary and serum cortisol concentrations after adrenocorticotropic hormone challenge in ewes. J Anim Sci.

[22] Schwinn AC, Knight CH, Bruckmaier RM, Gross JJ (2016). Suitability of saliva cortisol as a biomarker for hypothalamic–pituitary–adrenal axis activation assessment, effects of feeding actions, and immunostimulatory challenges in dairy cows. J Anim Sci.

[23] Hernandez CE, Thierfelder T, Svennersten-Sjaunja K (2014). Time lag between peak concentrations of plasma and salivary cortisol following a stressful procedure in dairy cattle. Acta Vet Scand.

[24] Goldhawk C, Bond G, Grandin T, Pajor E (2016). Behaviour of bucking bulls prior to rodeo performances and relation to rodeo and human activities. Appl Anim Behav Sci.

[25] Sánchez-Rodríguez HL, Vann RC, Youngblood RC (2013). Evaluation of pulsatility index and diameter of the jugular vein and superficial body temperature as physiological indices of temperament in weaned beef calves: Relationship with serum cortisol concentrations, rectal temperature, and sex. Livest Sci.

[26] Morrow CJ, Kolver ES, Verkerk GA, Matthews LR (2002). Fecal glucocorticoid metabolites as a measure of adrenal activity in dairy cattle. Gen Comp Endocrinol.

[27] Bousquet-Mélou A, Formentini E, Picard-Hagen N (2006). The adrenocorticotropin stimulation test: Contribution of a physiologically based model developed in horse for its interpretation in different pathophysiological situations encountered in man. Endocrinology.

[28] Geburt K, Piechotta M, König von Borstel U, Gauly M (2015). Influence of testosterone on the docility of German Simmental and Charolais heifers during behavior tests. Physiol Behav.

[29] Aurich J, Wulf M, Ille N (2015). Effects of season, age, sex, and housing on salivary cortisol concentrations in horses. Domest Anim Endocrinol.

[30] Olbrich D, Dittmar M (2012). The cortisol awakening response is related with PERIOD1 clock gene expression in older women. Exp Gerontol.

[31] Xavier SS, Allwin B, Vedaminckam S, Kalyaan US (2015). Changes in the fecal concentrations of cortisol metabolites in response to stress in Gaurs (*Bos gaurus*) in three wildlife regions with respect to climatic change and conflict occurence—A non-invasive study. Int J Appl Pure Sci Agric.

[32] Huber S, Palme R, Arnold W (2003). Effects of season, sex and sample collection on concentrations of faecal cortisol metabolites in red deer (*Cervus elaphus*). Gen Comp Endocrinol.

[33] Forkman B, Boissy A, Meunier-Salaün MC, Canali E, Jones RB (2007). A critical review of fear tests used on cattle, pigs, sheep, poultry and horses. Physiol Behav.

[34] Munksgaard L, De Passillé AM, Rushen J, Thodberg K, Jensen MB (1997). Discrimination of people by dairy cows based on handling. J Dairy Res.

[35] Dunn CS (1990). Stress reactions of cattle undergoing ritual slaughter using two methods of restraint. Vet Rec.

